# Changes in Pharyngeal Airway Space and Craniocervical Angle after Anterior Bimaxillary Subapical Osteotomy

**DOI:** 10.1155/2021/9978588

**Published:** 2021-08-10

**Authors:** Jung-Hsuan Cheng, Chun-Feng Chen, Ping-Ho Chen, Kun-Jung Hsu, Han-Sheng Chen, Chun-Ming Chen

**Affiliations:** ^1^Department of Orthodontics, Kaohsiung Medical University Hospital, Kaohsiung, Taiwan; ^2^Department of Oral and Maxillofacial Surgery, Kaohsiung Veterans General Hospital, Kaohsiung, Taiwan; ^3^School of Dentistry, College of Dental Medicine, Kaohsiung Medical University, Kaohsiung, Taiwan; ^4^Department of Dental Technology, Shu-Zen Junior College of Medicine and Management, Kaohsiung, Taiwan; ^5^Department of Dentistry, Kaohsiung Municipal Ta-Tung Hospital, Kaohsiung, Taiwan; ^6^Dental Department, Kaohsiung Municipal Siaogang Hospital, Kaohsiung, Taiwan; ^7^Department of Oral and Maxillofacial Surgery, Kaohsiung Medical University Hospital, Kaohsiung Medical University, Taiwan

## Abstract

**Purpose:**

This study explored the effects of genioplasty (Gep) and anterior subapical osteotomy of the maxilla and mandible (ASOMx+ASOMd) on the pharyngeal airway dimensions of patients with bimaxillary protrusion (BiP).

**Method:**

Thirty-two patients were divided into 2 groups. Group 1 received ASOMx+ASOMd, and group 2 received ASOMx+ASOMd+Gep. The cephalograms of the patients were collected before surgery and 2 months after surgery. Changes in the landmarks, related cephalometric angles (gonial, SN-GoGn, *Y*-axis, and SN-C2C4 angles), and 2 pharyngeal airway dimensions (uvulo-pharyngeal airway [UOP] and tongue–pharyngeal airway [TOP]) were analyzed.

**Results:**

Before surgery, the parameters (incisor superius, incisor inferius, menton, most superior and anterior point of the hyoid bone, tip of the uvula, inferoanterior point on the second cervical vertebra, and inferoanterior point on the fourth cervical vertebra) and measured angles (SNA, SNB, ANB, gonial, SN-GoGn, *Y*-axis, and C4C2-SN) of both groups showed no significant differences. Following ASOMx, the patients in groups 1 and 2 exhibited a setback by 7.0 and 6.6 mm, respectively. After ASOMd, groups 1 and 2 exhibited 4.9 and 5.3 mm setbacks, respectively. No significant difference in the amount of setback was observed between groups 1 and 2. The postoperative horizontal and vertical positions of Me in group 2 were significantly forward by 6.1 mm and upward by 1.5 mm, respectively. Regarding pharyngeal airway dimensions, TOP was decreased in group 1 (1.7 mm) and group 2 (1.3 mm). In the postoperative Pearson correlation coefficient test, the horizontal and vertical positions of Me showed no significant correlation with TOP in both groups. Therefore, Gep did not prevent the reduction of TOP in group 2.

**Conclusion:**

After bimaxillary anterior subapical osteotomy, the TOP of patients with BiP was decreased, and this situation was unavoidable, regardless of whether Gep was performed.

## 1. Introduction

Bimaxillary protrusion (BiP) is a facial deformity associated with the anterior segments of the maxilla and mandible. This condition is characterized by severe protrusion of the maxillary and mandibular dental arches and is frequently accompanied by underdevelopment of the chin. Patients with BiP exhibit a convex facial profile and experience difficulty closing their lips; consequently, they must flex their facial muscles to close their lips, causing mentalis muscle strain [1–3]. A gummy smile or toothy face is a common clinical characteristic and the most frequent chief complaint of such patients. Patients with BiP typically exhibit properly aligned teeth, with normal posterior occlusion, although some patients might exhibit slightly crowded teeth. The deformity in the facial profiles of such patients affects them psychologically and causes them to develop an introverted personality or to feel inferior or angry, which in turn affects their work and social interactions.

Depending on symptom severity, the clinical treatment for BiP involves orthodontic correction alone or in combination with orthognathic surgery. Bimaxillary anterior subapical osteotomy (ASO) is an established surgical technique for the treatment of BiP. Although orthognathic surgery can improve the facial profile of patients with BiP, several studies [4–6] have demonstrated that the surgery causes mandibular setback and affects the organization of tissues. In addition, the bimaxillary setback procedure considerably reduces the pharyngeal airway space and lowers the position of the tongue and hyoid, thereby changing the position of the head. Therefore, this study investigated the effects of maxillary ASO (ASOMx) and mandibular ASO (ASOMd) with or without genioplasty (Gep) on dental position, pharyngeal airway space, and head position in patients with BiP. The null hypothesis was that the postoperative tongue–pharyngeal airway dimension would not differ significantly between group 1 (without Gep) and group 2 (with Gep).

## 2. Material and Method

**Figure 1:**
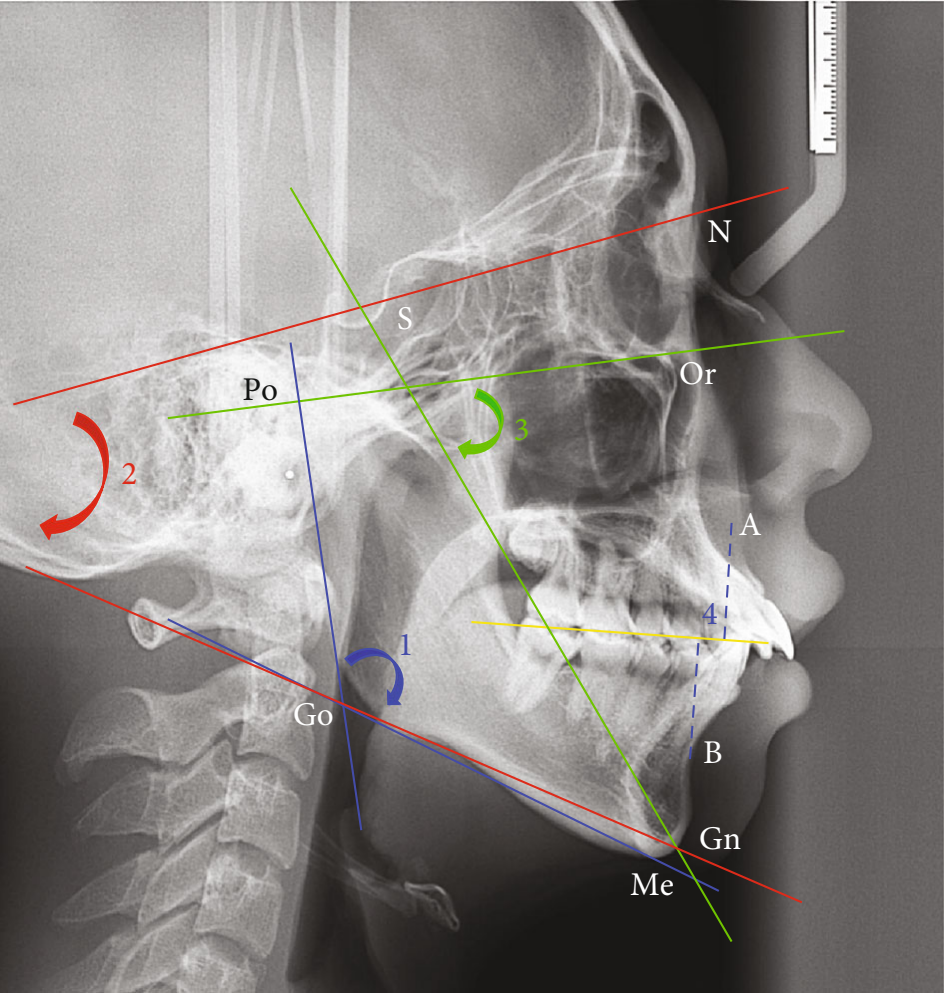
Cephalometric landmarks: S: sella; N: nasion; Or: orbitale; Po: porion; A point; B point; Gn: gnathion; Me: menton; Go: gonion. The following measurements: (1) blue color: gonial angle (lines tangent to the posterior border of the ramus and Go-Me plane), (2) red color: SN-GoGn angle (angle formed by lines S-N and Go-Gn), (3) green color: *Y*-axis angle (angle between S-Gn and Or-Po plane), and (4) Wits appraisal (mm).

**Figure 2:**
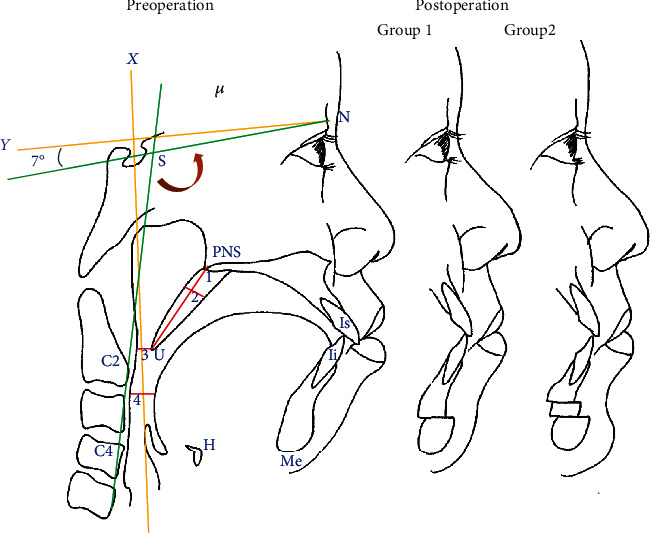
Preoperation and postoperation (group 1 and group 2) in patient with bimaxillary protrusion. *X*-axis (horizontal axis): a line through nasion 7° up from SN line. *Y*-axis (vertical axis): a line through sella (S) perpendicular to the *X*-axis. The measurements: (1) length of the soft palate and (2) width of the soft palate. Pharyngeal airway space: (3) UOP and (4) TOP craniocervical angle: C2C4-SN angle. Group 1 received ASOMx+ASOMd, and group 2 received ASOMx+ASOMd+GeP.

This study included 32 patients with BiP who received treatment at the Department of Oral and Maxillofacial Surgery, Kaohsiung Medical University. The inclusion criteria of this study were as follows: (1) BiP without deformed lips prior to surgery and (2) no other facial injuries or etiology. The patients were divided into 2 groups. Group 1 underwent ASOMx+ASOMd, and group 2 underwent ASOMx+ASOMd+Gep (Figures 1 and 2). Group 1 (mean age: 28.9, ranged from 19 to 43) comprised 14 female and 2 male patients, and group 2 (mean age: 24.8, ranged from 16 to 33) included 13 female and 3 male patients. Groups 1 and 2 did not differ significantly (*p* = 0.061) in terms of age by the Student *t*-test. According to the classification of skeletal patterns (class I, 0° < ANB < 4°; class II, ANB ≥ 4°; class III, ANB ≤ 0°), group 1 had 11 class II patients and 5 class I patients, and group 2 had 14 class II patients and 2 class I patients.

All patients received the traditional fixed orthodontic appliance (OPAK system bracket with a 0.022 in × 0.028 in slot [Tomy Co., L, Tokyo, Japan]). In group 1, the mean durations of pre- and postsurgical orthodontic treatment were 5.8 and 13.5 months, respectively. In group 2, the mean durations of pre- and postsurgical orthodontic treatment were 5.3 and 14.2 months, respectively. The mean total orthodontic treatment time of group 1 and group 2 were 19.3 and 19.5 months, respectively. The 4 first premolars were extracted during the surgical procedure, and then, ASOMx+ASOMd was performed through the extraction spaces.

The cephalograms of each patient were collected preoperatively and 2 months postoperatively. The following landmarks were recorded: sella (S), nasion (N), posterior nasal spine (PNS), incisor superius (Is), incisor inferius (Ii), gnathion (Gn), menton (Me), gonion (Go), tip of the uvula (U), inferoanterior point on the second cervical vertebra (C2), inferoanterior point on the fourth cervical vertebra (C4), and most superior and anterior point of the hyoid bone (H). The following distances were measured: length of the soft palate (i.e., the distance between U and PNS [SPL]), the widest distance of the soft palate (SPW), and the Wits appraisal (mm). The following angles were measured: lines tangent to the posterior border of the ramus and Go-Me plane (gonial angle), angle formed by lines S-N and Go-Gn (SN-GoGn angle), angle between S-Gn and the Frankfort horizontal plane (*Y*-axis angle), and craniocervical angle (i.e., angle between C2C4 line and SN line [SN-C2C4 angle]). The pharyngeal airways were also measured as follows: (1) uvula pharyngeal airway (i.e., distance between the horizontal plane through U intersecting posterior pharyngeal wall [UOP]) and (2) shortest distance from the posterior tongue to the pharyngeal wall (TOP).

The process of cephalometric landmark identification was performed twice by the author. Subsequently, the calculated intraobserver reliability (correlation coefficient > 0.900, *p* < 0.001) was determined to be acceptable. The changes in surgical landmarks were collected for statistical analyses (SPSS version 20, IBM Corporation, Armonk, NY, USA), including the calculation of mean and standard deviation values. Student's *t*-test was used with a 95% confidence level to test the statistical significance. The Pearson correlation coefficient test was performed to compare the correlations between the variables and pharyngeal airway dimensions. The correlation strength was derived as the absolute value of the ratio of the compared variables: very weak (0-0.19), weak (0.20-0.39), moderate (0.40-0.59), strong (0.60-0.79), and very strong (0.80-1.0).

## 3. Results

The preoperative characteristics of group 1 and group 2 are presented in Table 1. Regarding the horizontal and vertical position of several landmarks (Is, Ii, Me, H, U, C2, and C4), no significant difference was noted between group 1 and group 2. All measured angles (SNA, SNB, ANB, gonial, SN-GoGn, *Y*-axis, and C4C2-SN) exhibited no significant difference between groups 1 and 2 (Table 2). Therefore, the baseline vertical and horizontal patterns did not differ significantly between groups 1 and 2. Furthermore, the SPL and pharyngeal airway space (UOP and TOP) did not differ significantly between groups 1 and 2. The postoperative results obtained for groups 1and 2 are presented in Tables 3 and 4. Is and Ii were significantly set back by 7.0 and 4.9 mm, respectively, in group 1 and by 6.6 and 5.3 mm, respectively, in group 2 (Table 3). Me was significantly advanced forward by 6.1 mm in group 2. However, the postoperative position of the landmarks (Is, Ii, H, U, C2, and C4) did not differ significantly between groups 1 and 2. Postoperative changes in H revealed no significant difference between groups 1 and 2, indicating that the advancement of Gep exerted no significant effect on the H position.

As presented in Table 4, SNA and SNB were significantly decreased in group 1 after surgery. The measured angles (SNA, SNB, ANB, gonial, and SN-GoGn) were significantly decreased in group 2 (Table 4). In the intergroup comparison, the gonial angle in group 2 was significantly decreased relative to that in group 1. The increase in SPL was nonsignificant between groups 1 and 2. Concerning changes in the pharyngeal airway space, UOP and TOP were significantly reduced by 2.2 and 1.7 mm, respectively, in group 1. Changes in UOP and TOP in group 2 were nonsignificant. The SN-C2C4 angle of the 2 groups exhibited no significant differences after surgery.

Table 5 lists postoperative changes in landmarks and pharyngeal airways derived in the Pearson correlation test. In group 1, UOP and TOP exhibited no significant correlation with the landmarks (Is, Ii, Me, H, U, C2, and C4). In group 2, horizontal U exhibited a significant strong positive correlation with UOP (*r* = 0.770) and significant moderate correlation with TOP (*r* = 0.593). Vertical U exhibited a significant strong negative correlation with TOP (*r* = −0.726) in group 2.  Table 6 lists the results of the Pearson correlation coefficient test between pharyngeal airways and related measured angles. In group 1, the gonial angle exhibited a significant moderate positive correlation (*r* = 0.533) with TOP. In group 2, UOP had a moderate positive correlation (*r* = 0.504) with the ANB angle and a significant strong negative correlation (*r* = −0.634) with SPL. TOP showed a significant moderate negative correlation (*r* = −0.560) with SPL. Furthermore, significant positive correlations were observed between UOP and TOP in both groups (group 1: *r* = 0.559 and group 2: *r* = 0.622). The horizontal and vertical positions of Me were not significantly correlated with TOP in both groups. Accordingly, the null hypothesis was accepted, demonstrating that Gep advancement is not significantly correlated with changes in TOP.

## 4. Discussion

The prevalence of facial deformity and malocclusion varies considerably within different races. Farrow et al. [7] observed that black Americans differ significantly from white Americans in terms of dental, skeletal, and soft tissue parameters. Drummond [8] compared white Americans with black Americans and discovered that black patients exhibited bimaxillary dental protrusion, a steep mandibular plane, and anterior placement of the maxilla. Boeck et al. [9] surveyed the occurrence of skeletal malocclusions in Brazilian patients with dentofacial deformities and observed a low incidence (7%) of BiP in the Caucasians. Isiekwe [10] reported a 20% prevalence of BiP in Nigeria, with 75% having a skeletal class I jaw relationship. Sundareswaran and Kizhakool [11] examined malocclusion in 13–15-year-old adolescents in southern India and reported a 21.3% prevalence of BiP.

The pharynx is a muscular channel with a wide top and a narrow bottom. The top of the pharynx is connected with the cranial basis, and the bottom is located near the sixth cervical vertebra. The front wall of the pharynx is not completely sealed and is connected to the nasopharynx, oropharynx, and laryngopharynx. The posterior wall of the pharynx is composed of loose connective tissues that are attached to the prevertebral fascia. From top to bottom, the pharynx is divided into 3 parts: the nasopharynx, oropharynx, and laryngopharynx (or hypopharynx). The nasopharynx and oropharynx are separated by the palate, and the oropharynx and laryngopharynx are separated by the epiglottis [12, 13]. Both food and air pass through the pharyngeal airway, where the digestive tract and respiratory tract intersect. Therefore, the pharynx is crucial to swallowing and respiratory functions and affects respiratory defense mechanisms, middle ear pressure regulation, and auditory functioning. The pharynx also serves as a resonance cavity that can adjust its size when required through the raising and lowering of the dorsum and soft palate.

BiP presents a convex facial profile and can be corrected by orthodontic treatment alone or in combination with orthognathic surgery. By contrast, maxillary deficiency can be treated using various nonsurgical approaches in growing patients. Jamilian et al. [14] used a tongue appliance to push the maxilla into a forward position in growing patients with maxillary deficiency. Jamilian et al. [14] evaluated the effects of treatment with a maxillary protraction appliance (tongue appliance) on upper airway dimensions and demonstrated that a tongue appliance does not affect sagittal airway dimensions but increases vertical airway dimensions within a short time. Pamporakis et al. [15] investigated the effects of rapid maxillary expansion (RME) and facemask (FM) use on pharyngeal airway space in growing patients with class III maxillary deficiency. After RME/FM treatment, a significant increase was observed in the maxillary sinus volume, whereas the increases in the volumes of the upper and lower pharyngeal airway space were nonsignificant. Tahmasbi et al. [16] compared the effects of 2 surgical methods, anterior maxillary segmental distraction (AMSD) versus conventional Le Fort I osteotomy, on cephalometric changes in the velopharyngeal area of patients with cleft lip and palate. They observed that AMSD could improve the facial profile to a level almost similar to that achieved by conventional Le Fort I advancement; although a significant decrease was observed in the nasopharyngeal area, no increase was noted in the velopharyngeal sphincter. However, conventional Le Fort I maxillary advancement could be effective in increasing the pharyngeal airway space.

Recently, the development of theories related to surgical techniques and the advances in hypotensive anesthesia technology have increased the frequency of orthognathic surgery for the treatment of facial deformities. Orthognathic surgery is no longer limited to patients with severe conditions; those who seek efficient treatment outcome or who hope to reduce treatment time also receive orthognathic surgery-assisted treatment. However, such surgery displaces both jaws, thereby altering the airway space. Scholars [17–19] have suggested that mandibular advancement surgery causes the forward movement of the hyoid bone, whereas mandibular setback surgery results in the backward movement of the hyoid bone. Studies [18, 19] have also reported that mandibular setback surgery causes the backward movement of the tongue, leading to the narrowing of the airway space.

The ANB angle and Wits appraisal are common cephalometric parameters used in the interpretation of the anteroposterior jaw relationship. The Wits appraisal is a valuable linear cephalometric measurement used in evaluating the anteroposterior relationship of the anterior bimaxillary apical bases. The Wits appraisal is also commonly applied in the diagnosis of the severity or degree of anteroposterior jaw disharmony. Before and after surgery, no significant difference in the ANB angle and Wits appraisal was observed in both group 1 and group 2. Gonial angle is a cephalometric analysis used to both predict the growth pattern and infer the rotation of the mandible. SN-GoGn angle is used to assess the mandibular vertical growth and determine the direction of mandibular growth rotation. *Y*-axis angle is also an indicator of mandibular growth direction. In the present study, we used gonial angle, SN-GoGn angle, and *Y*-axis angle to evaluate the growth pattern and the rotation of the mandible. Both groups showed similar facial patterns.

In the ASOMx procedure, the 4 first premolars are removed to achieve setback of the anterior maxillary segment. In this study, ASOMx resulted in the setback and upward displacement of the Is position. Anatomically, ASOMx causes changes to soft tissues mainly in the upper lips and nose and exerts a minimal effect on the pharyngeal airway, especially in the UOP. Before surgery, the Is positions of group 1 were more anterior than those of group 2. The amount of setback in group 1 (7.0 mm) was larger than that in group 2 (6.6 mm). Moreover, preoperative UOP did not differ significantly between groups 1 and 2. The postoperative intragroup comparison revealed that UOP was significantly reduced by 2.2 mm in group 1 and was nonsignificantly increased by 0.2 mm in group 2. This finding can be because group 1 had a larger setback in Is without Gep for chin advancement.

Before surgery, the Ii positions of groups 1 and 2 did not differ significantly. However, group 2 exhibited more chin deficiency than group 1 did. Preoperatively, the Me position of group 2 was 4.9 mm behind that of group 1. After surgery, the Ii setback distance of group 1 was 4.9 mm, which was smaller than that of group 2 (5.3 mm). Consequently, group 2 required Gep to advance Me by 6.1 mm. After surgery, the horizontal Me positions of groups 1 and 2 were 55.9 and 56.7 mm, respectively, and the vertical Me distances were 133.0 and 130.9 mm, respectively. Therefore, the Me positions of the 2 groups differed nonsignificantly after surgery. The postoperative H position of group 2 was forward by 1.5 mm, indicating that Gep affected the H position. However, this finding did not reach statistical significance. After surgery, the SPL of the 2 groups increased. This might be attributable to the setback of the tongue following ASOMd, which stretched the palatal arch muscle and thereby increased the soft palate length. Because the middle pharyngeal constrictor muscle is proximally attached to the hyoid bone, the geniohyoid muscle passes from the chin to the hyoid bone. Therefore, the postoperative H of group 2 was moved forward by 1.5 mm and upward by 1.6 mm through Gep advancement. A significant postoperative reduction in UOP was found in group 1 (without Gep). Chin advancement (Gep) affected the attached muscles, including the hyoglossus, genioglossus, geniohyoid, and mylohoid. Thus, TOP could be changed after Gep. The extent of TOP reduction in group 1 (1.7 mm) was larger than that in group 2 (1.3 mm). However, the postoperative TOP of group 2 decreased by only 0.4 mm through Gep. Although Gep prevented the reduction of UOP and TOP, the corresponding result did not reach statistical significance.

This study also examined whether the relative positions of the head and cervical vertebrae are changed following surgery. Clinical observation showed that prior to surgery, the head positions of the patients tilted slightly downward compared with the healthy patients. This posture enables such patients to conceal the protruded facial profile caused by BiP. After surgery, setback and lowering of the C2 landmark were observed in both groups. This might have been caused by the narrowing of the airway space, which would prompt patients to naturally adjust their head positions to improve respiratory function [20]. Accordingly, the patients' head positions moved slightly backward and tilted upward. Nevertheless, the position of C2 and C4 changed nonsignificantly. Another notable physiological change among the patients was the increase in SN-C2C4 angle, which was due to reductions in the UOP and TOP. This was evident after surgery, as patients naturally tilted their heads upward to breathe more easily and to accommodate the reductions in the UOP and TOP.

Pearson correlation analysis was performed to determine the correlation of postoperative changes in UOP and TOP with landmarks. No significant correlations were observed in group 1. However, changes in the horizontal and vertical positions of U showed moderate positive and strong negative correlations with TOP, respectively. This finding demonstrates that Gep moved hyoid bone anterosuperiorly and resulted in a lower reduction in postoperative TOP. However, the advancement of Me by Gep in group 2 presented no significant correlation with UOP or TOP. Gep did not prevent the reduction of TOP.

## 5. Conclusion

Regardless of whether they receive Gep, patients with BiP experience a reduction of the pharyngeal airway space following ASOMx+ASOMd. We observed that the reduction of the pharyngeal airway space is caused primarily by the setback of the mandible. After surgery, such patients must adjust their head position as required by natural physiological function. This increases the SN-C2C4 angle, allowing patients to breathe more easily. This indicates that mandibular setback surgery affects the pharyngeal airway space and head position of patients with BiP.

## Figures and Tables

**Table 1:** Preoperative characteristics in both groups.

Variables	Group 1		Group 2		Intergroup comparison
	Mean	SD	Mean	SD	*p* value
Horizontal					
Is	80.6	8.45	79.0	4.11	0.517
Ii	75.9	7.53	74.9	4.35	0.661
Me	55.5	10.13	50.6	6.07	0.139
H	14.0	8.47	11.8	9.64	0.551
U	0.3	6.61	-0.7	4.96	0.612
C2	-20.1	7.30	-20.6	7.45	0.833
C4	-25.7	11.09	-26.1	10.22	0.919
Vertical					
Is	91.6	4.13	92.0	4.30	0.774
Ii	88.1	4.44	88.4	3.65	0.857
Me	131.3	5.17	132.4	4.26	0.579
H	116.9	6.95	118.9	11.32	0.631
U	82.9	3.49	82.9	6.34	1.000
C2	95.4	5.35	95.1	7.05	0.876
C4	130.8	6.04	131.1	9.73	0.939

Group 1: ASO; group 2: ASO+Gep. ^∗^Intergroup comparison: statistically significant, *p* < 0.05.

**Table 2:** Preoperative pharyngeal airway-related value in both groups.

Variables	Group 1		Group 2		Intergroup comparison
	Mean	SD	Mean	SD	*p* value
SNA	85.1	2.76	86.1	2.41	0.210
SNB	80.4	3.58	80.2	3.27	0.821
ANB	4.6	2.58	6.0	1.73	0.068
Gonial	120.6	5.08	123.3	5.43	0.200
SN-GoGn	33.0	6.49	35.8	3.90	0.120
*Y*-axis	62.2	3.86	64.5	2.49	0.055
Wits appraisal	2.7	3.40	3.4	2.21	0.410
SN-C2C4	105.4	6.44	106.1	7.67	0.815
SPL	36.3	3.32	34.2	5.32	0.238
SPW	8.3	1.00	9.0	0.98	0.013^∗^
Pharyngeal airway					
UOP	12.4	2.19	10.9	3.34	0.123
TOP	11.9	3.27	12.1	2.96	0.811

Group 1: ASO; group 2: ASO+Gep. ^∗^Intergroup comparison: statistically significant, *p* < 0.05.

**Table 3:** Postoperative changes of characteristics in both groups.

Variables	Group 1			Group 2			Intergroup comparison
	Mean	SD	*p* value	Mean	SD	*p* value	*p* value
Horizontal							
Is	-7.0	3.19	<0.001^∗^	-6.6	2.0	<0.001^∗^	0.684
Ii	-4.9	2.78	<0.001^∗^	-5.3	2.0	<0.001^∗^	0.674
Me	0.4	2.83	0.603	6.1	2.3	<0.001^∗^	<0.001^∗∗^
H	-0.4	4.24	0.728	1.5	6.4	0.355	0.253
U	-3.2	4.45	0.01^∗^	-0.6	4.6	0.609	0.091
C2	0.0	3.10	0.968	2.0	6.1	0.209	0.189
C4	-0.6	4.65	0.636	1.9	7.6	0.338	0.209
Vertical							
Is	-1.3	3.82	0.190	-1.9	5.5	0.180	0.743
Ii	0.1	4.54	0.957	-0.4	4.3	0.691	0.764
Me	0.7	4.11	0.495	-1.5	2.6	0.035^∗^	0.134
H	1.7	5.19	0.205	-1.6	5.4	0.251	0.130
U	-0.9	3.96	0.358	1.2	2.5	0.081	0.100
C2	1.2	4.53	0.299	0.9	4.8	0.446	0.879
C4	1.4	6.32	0.387	-0.5	3.8	0.615	0.394

Group 1: ASO; group 2: ASO+Gep. ^∗^Intragroup comparison: statistically significant, *p* < 0.05. ^∗∗^Intergroup comparison: statistically significant, *p* < 0.05.

**Table 4:** Postoperative changes of pharyngeal airway in both groups.

Variables	Group 1			Group 2			Intergroup comparison
	Mean	SD	*p* value	Mean	SD	*p* value	*p* value
SNA	-2.7	2.44	0.001^∗^	-3.5	2.07	<0.001^∗^	0.387
SNB	-2.0	1.88	0.001^∗^	-2.0	1.88	0.001^∗^	0.966
ANB	-0.6	1.88	0.226	-1.5	1.55	0.001^∗^	0.206
Gonial	1.4	3.08	0.088	-3.8	3.61	0.001^∗^	<0.001^∗∗^
SN-GoGn	-1.2	3.29	0.180	-2.2	2.28	0.002^∗^	0.341
*Y*-axis	0.5	3.08	0.526	-1.0	2.38	0.103	0.050
Wits appraisal	-1.0	3.47	0.248	-1.4	1.84	0.010^∗^	0.764
SN-C2C4	1.4	3.52	0.131	0.3	5.99	0.838	0.556
SPL	1.4	3.35	0.106	1.2	3.09	0.131	0.840
SPW	0.8	1.38	0.032^∗^	-1.0	1.13	0.002^∗^	<0.001^∗∗^
Pharyngeal airway							
UOP	-2.2	2.42	0.003^∗^	0.2	3.08	0.794	0.028^∗∗^
TOP	-1.7	2.56	0.017^∗^	-1.3	2.99	0.116	0.600

Group 1: ASO; group 2: ASO+Gep. ^∗^Intragroup comparison: statistically significant, *p* < 0.05. ^∗∗^Intergroup comparison: statistically significant, *p* < 0.05.

**Table 5:** Pearson's correlation coefficient test between pharyngeal airways and landmarks.

Variables	Group 1		Group 2	
	UOP	TOP	UOP	TOP
Horizontal				
Is	0.304	0.237	0.020	-0.328
Ii	0.214	0.344	0.021	0.131
Me	0.323	0.188	0.022	0.112
H	0.222	-0.100	0.344	0.245
U	0.434	0.034	0.770^∗^	0.593^∗^
C2	0.221	-0.146	0.178	0.181
C4	0.008	-0.143	0.036	-0.049
Vertical				
Is	-0.064	-0.222	-0.057	-0.468
Ii	-0.108	-0.197	-0.072	-0.330
Me	-0.039	-0.284	0.231	-0.275
H	-0.047	-0.416	-0.181	-0.495
U	0.085	-0.025	-0.289	-0.726^∗^
C2	0.311	-0.055	0.217	0.157
C4	0.156	-0.105	0.020	-0.081

^∗^Statistically significant, *p* < 0.05.

**Table 6:** Pearson's correlation coefficient test between pharyngeal airways and related measured angles.

Variables	Group 1		Group 2	
	UOP	TOP	UOP	TOP
SNA	0.005	-0.135	0.444	0.468
SNB	-0.027	0.092	-0.028	0.311
ANB	0.051	-0.258	0.504^∗^	0.352
Gonial	0.161	0.533^∗^	0.357	0.115
SN-GoGn	0.050	0.023	0.236	0.107
*Y*-axis	0.027	-0.055	0.219	0.190
Wits appraisal	0.347	0.356	-0.009	0.167
SN-C2C4	0.178	0.150	0.203	0.260
SPL	-0.158	-0.191	-0.634^∗^	-0.560^∗^
SPW	0.239	-0.211	-0.085	0.026
UOP	1	0.559^∗^	1	0.622^∗^
TOP	0.559^∗^	1	0.622^∗^	1

^∗^Statistically significant, *p* < 0.05.

## Data Availability

The data used to support the findings of this study are included within the article and are also available from the corresponding author upon request.

## References

[1] Bills D. A., Handelman C. S., BeGole E. A. (2005). Bimaxillary dentoalveolar protrusion: traits and orthodontic correction. *The Angle Orthodontist*.

[2] Carter N. E., Slattery D. A. (1988). Bimaxillary proclination in patients of Afro-Caribbean origin. *British Journal of Orthodontics*.

[3] Keating P. J. (1985). Bimaxillary protrusion in the Caucasian: a cephalometric study of the morphological features. *British Journal of Orthodontics*.

[4] Tseng Y. C., Hsiao S. Y., Cheng J. H., Hsu K. J., Chen C. M. (2020). Postoperative skeletal stability and pharyngeal airway: counterclockwise versus clockwise rotation during mandibular setback surgery. *BioMed Research International*.

[5] An J. H., Park S. B., Choi Y. K., Lee S. H., Kim K. B., Kim Y. I. (2019). Cone-beam computed tomography evaluation of pharyngeal airway space changes after bimaxillary orthognathic surgery in patients with class III skeletal deformities: a 6-year follow-up study. *Journal of Oral and Maxillofacial Surgery*.

[6] Lee S. T., Park J. H., Kwon T. G. (2019). Influence of mandibular setback surgery on three-dimensional pharyngeal airway changes. *International Journal of Oral and Maxillofacial Surgery*.

[7] Farrow A. L., Zarrinnia K., Azizi K. (1993). Bimaxillary protrusion in black Americans--an esthetic evaluation and the treatment considerations. *American Journal of Orthodontics and Dentofacial Orthopedics*.

[8] Drummond R. A. (1968). A determination of cephalometric norms for the Negro race. *American Journal of Orthodontics*.

[9] Boeck E. M., Lunardi N., Pinto A. S., Pizzol K. E., Boeck Neto R. J. (2011). Occurrence of skeletal malocclusions in Brazilian patients with dentofacial deformities. *Brazilian Dental Journal*.

[10] Isiekwe M. (1990). Prevalence of bimaxillary protrusion in a Nigerian population. *Odonto-Stomatologie Tropicale*.

[11] Sundareswaran S., Kizhakool P. (2019). Prevalence and gender distribution of malocclusion among 13-15-year-old adolescents of Kerala, South India. *Indian Journal of Dental Research*.

[12] Berkovitz B. K. B., Moxham B. J. (1988). The Mouth, Palate and Pharynx. *A Textbook of Head and Neck Anatomy*.

[13] Lloyd E. (1988). *Dubrul the Oral Viscera: Sicher and Dubrul’s Oral Anatomy*.

[14] Jamilian A., Showkatbakhsh R., Borna N., Perillo L. (2014). The effects of maxillary protrusion on pharyngeal airway dimensions. *Journal of Dental Problems and Solutions*.

[15] Pamporakis P., Nevzatoğlu Ş., Küçükkeleş N. (2014). Three-dimensional alterations in pharyngeal airway and maxillary sinus volumes in class III maxillary deficiency subjects undergoing orthopedic facemask treatment. *The Angle Orthodontist*.

[16] Tahmasbi S., Jamilian A., Showkatbakhsh R., Pourdanesh F., Behnaz M. (2018). Cephalometric changes in nasopharyngeal area after anterior maxillary segmental distraction versus Le Fort I osteotomy in patients with cleft lip and palate. *European Journal of Dentistry*.

[17] Marşan G., Vasfi Kuvat S., Öztaş E., Cura N., Süsal Z., Emekli U. (2009). Oropharyngeal airway changes following bimaxillary surgery in class III female adults. *Journal of Cranio-Maxillo-Facial Surgery*.

[18] Kawamata A., Fujishita M., Ariji Y., Ariji E. (2000). Three-dimensional computed tomographic evaluation of morphologic airway changes after mandibular setback osteotomy for prognathism. *Oral Surgery, Oral Medicine, Oral Pathology, Oral Radiology, and Endodontics*.

[19] Greco J. M., Frohberg U., Van Sickels J. E. (1990). Long-term airway space changes after mandibular setback using bilateral sagittal split osteotomy. *International Journal of Oral and Maxillofacial Surgery*.

[20] Chen C. M., Lai S., Chen K. K., Lee H. E. (2015). Correlation between the pharyngeal airway space and head posture after surgery for mandibular prognathism. *BioMed Research International*.

